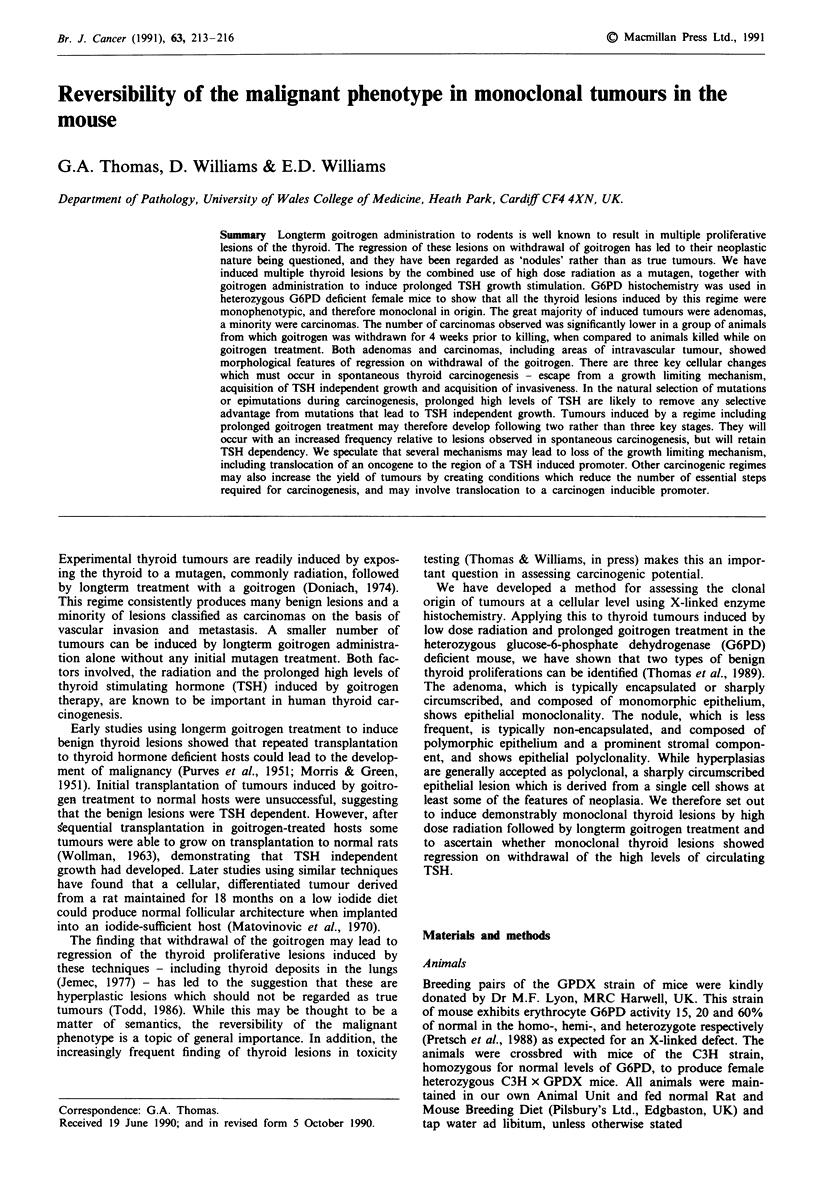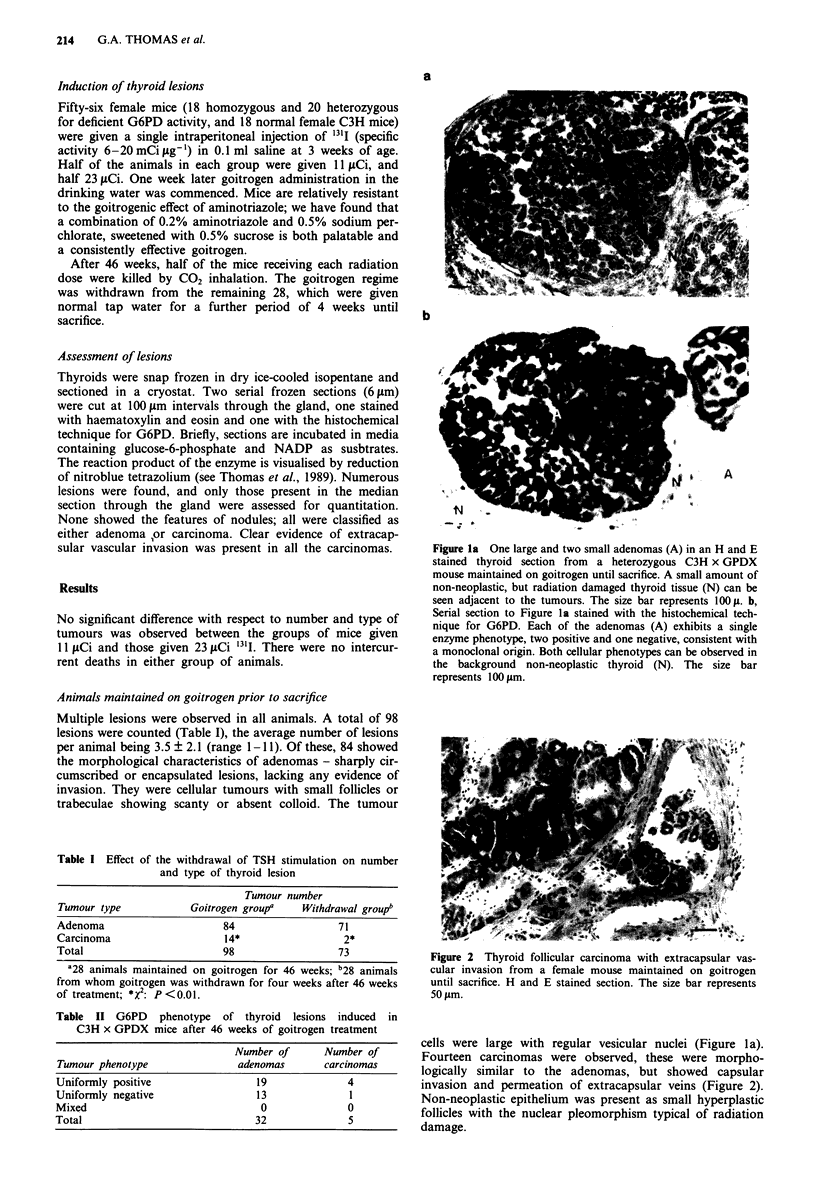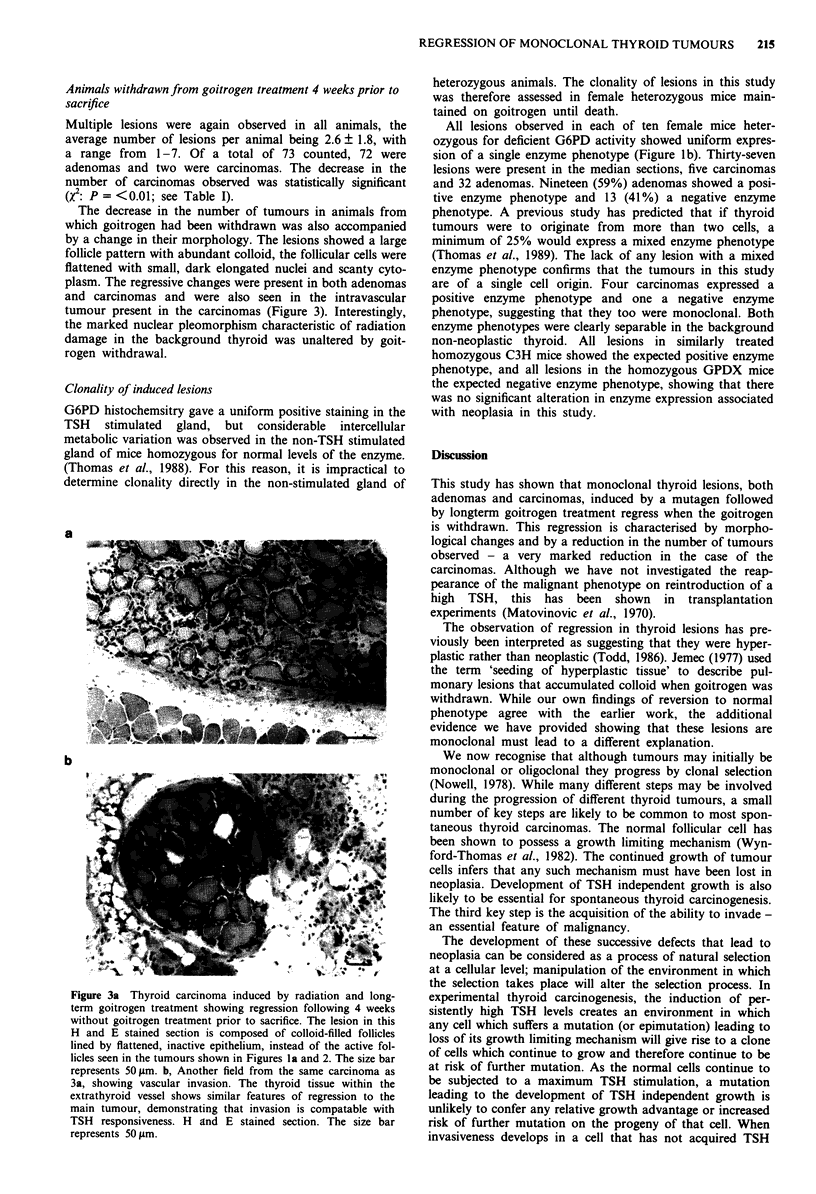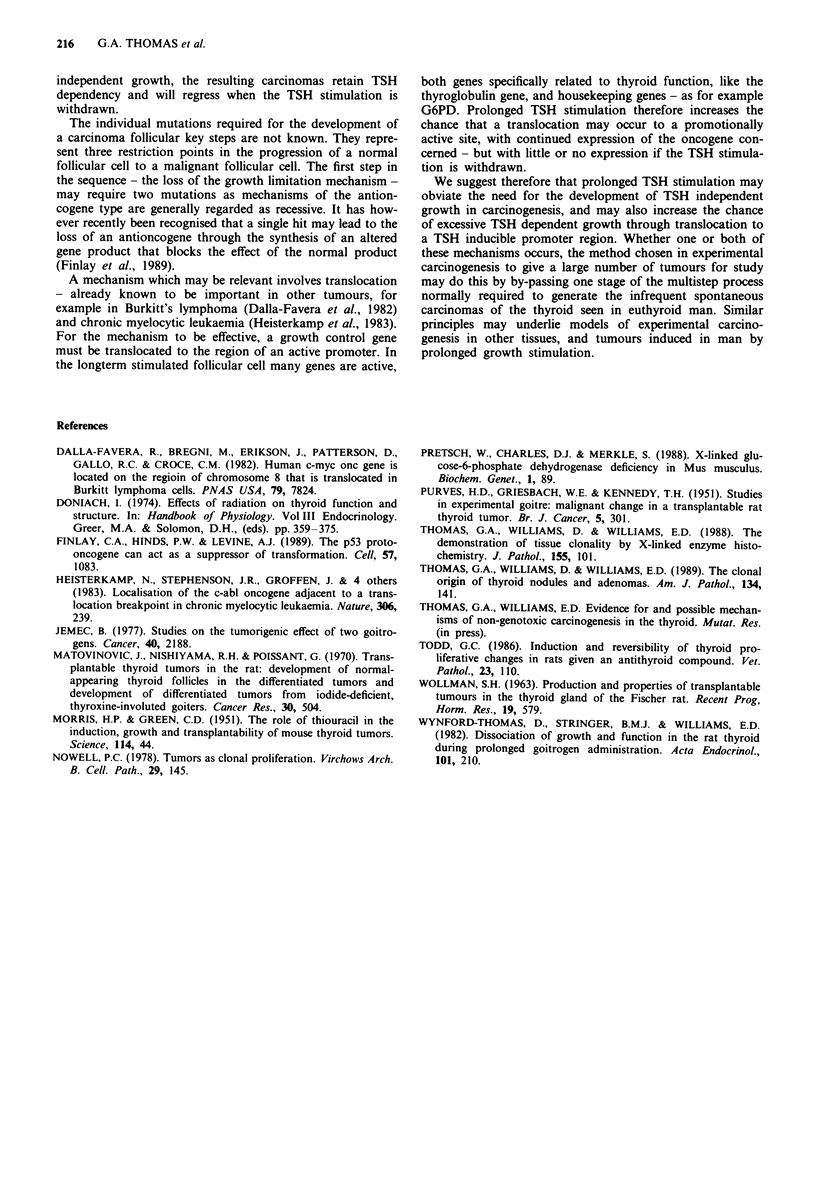# Reversibility of the malignant phenotype in monoclonal tumours in the mouse.

**DOI:** 10.1038/bjc.1991.51

**Published:** 1991-02

**Authors:** G. A. Thomas, D. Williams, E. D. Williams

**Affiliations:** Department of Pathology, University of Wales College of Medicine, Heath Park, Cardiff, UK.

## Abstract

**Images:**


Br. J. Cancer (1991), 63, 213-216 © Macmillan Press Ltd., 1991

### Reversibility of the malignant phenotype in monoclonal tumours in the mouse

G.A. Thomas, D. Williams & E.D. Williams

*Department of Pathology, University of Wales College of Medicine, Heath Park, Cardiff CF4 4XN, UK.*

**Summary** Longterm goitrogen administration to rodents is well known to result in multiple proliferative lesions of the thyroid. The regression of these lesions on withdrawal of goitrogen has led to their neoplastic nature being questioned, and they have been regarded as 'nodules' rather than as true tumours. We have induced multiple thyroid lesions by the combined use of high dose radiation as a mutagen, together with goitrogen administration to induce prolonged TSH growth stimulation. G6PD histochemistry was used in heterozygous G6PD deficient female mice to show that all the thyroid lesions induced by this regime were monophenotypic, and therefore monoclonal in origin. The great majority of induced tumours were adenomas, a minority were carcinomas. The number of carcinomas observed was significantly lower in a group of animals from which goitrogen was withdrawn for 4 weeks prior to killing, when compared to animals killed while on goitrogen treatment. Both adenomas and carcinomas, including areas of intravascular tumour, showed morphological features of regression on withdrawal of the goitrogen. There are three key cellular changes which must occur in spontaneous thyroid carcinogenesis - escape from a growth limiting mechanism, acquisition of TSH independent growth and acquisition of invasiveness. In the natural selection of mutations or epimutations during carcinogenesis, prolonged high levels of TSH are likely to remove any selective advantage from mutations that lead to TSH independent growth. Tumours induced by a regime including prolonged goitrogen treatment may therefore develop following two rather than three key stages. They will occur with an increased frequency relative to lesions observed in spontaneous carcinogenesis, but will retain TSH dependency. We speculate that several mechanisms may lead to loss of the growth limiting mechanism, including translocation of an oncogene to the region of a TSH induced promoter. Other carcinogenic regimes may also increase the yield of tumours by creating conditions which reduce the number of essential steps required for carcinogenesis, and may involve translocation to a carcinogen inducible promoter.

Experimental thyroid tumours are readily induced by exposing the thyroid to a mutagen, commonly radiation, followed by longterm treatment with a goitrogen (Doniach, 1974). This regime consistently produces many benign lesions and a minority of lesions classified as carcinomas on the basis of vascular invasion and metastasis. A smaller number of tumours can be induced by longterm goitrogen administration alone without any initial mutagen treatment. Both factors involved, the radiation and the prolonged high levels of thyroid stimulating hormone (TSH) induced by goitrogen therapy, are known to be important in human thyroid carcinogenesis.

Early studies using longerm goitrogen treatment to induce benign thyroid lesions showed that repeated transplantation to thyroid hormone deficient hosts could lead to the development of malignancy (Purves *et al.*, 1951; Morris & Green, 1951). Initial transplantation of tumours induced by goitrogen treatment to normal hosts were unsuccessful, suggesting that the benign lesions were TSH dependent. However, after sequential transplantation in goitrogen-treated hosts some tumours were able to grow on transplantation to normal rats (Wollman, 1963), demonstrating that TSH independent growth had developed. Later studies using similar techniques have found that a cellular, differentiated tumour derived from a rat maintained for 18 months on a low iodide diet could produce normal follicular architecture when implanted into an iodide-sufficient host (Matovinovic *et al.*, 1970).

The finding that withdrawal of the goitrogen may lead to regression of the thyroid proliferative lesions induced by these techniques - including thyroid deposits in the lungs (Jemec, 1977) - has led to the suggestion that these are hyperplastic lesions which should not be regarded as true tumours (Todd, 1986). While this may be thought to be a matter of semantics, the reversibility of the malignant phenotype is a topic of general importance. In addition, the increasingly frequent finding of thyroid lesions in toxicity testing (Thomas & Williams, in press) makes this an important question in assessing carcinogenic potential.

We have developed a method for assessing the clonal origin of tumours at a cellular level using X-linked enzyme histochemistry. Applying this to thyroid tumours induced by low dose radiation and prolonged goitrogen treatment in the heterozygous glucose-6-phosphate dehydrogenase (G6PD) deficient mouse, we have shown that two types of benign thyroid proliferations can be identified (Thomas *et al.*, 1989). The adenoma, which is typically encapsulated or sharply circumscribed, and composed of monomorphic epithelium, shows epithelial monoclonality. The nodule, which is less frequent, is typically non-encapsulated, and composed of polymorphic epithelium and a prominent stromal component, and shows epithelial polyclonality. While hyperplasias are generally accepted as polyclonal, a sharply circumscribed epithelial lesion which is derived from a single cell shows at least some of the features of neoplasia. We therefore set out to induce demonstrably monoclonal thyroid lesions by high dose radiation followed by longterm goitrogen treatment and to ascertain whether monoclonal thyroid lesions showed regression on withdrawal of the high levels of circulating TSH.

### Materials and methods

### *Animals*

Breeding pairs of the GPDX strain of mice were kindly donated by Dr M.F. Lyon, MRC Harwell, UK. This strain of mouse exhibits erythrocyte G6PD activity 15, 20 and 60% of normal in the homo-, hemi-, and heterozygote respectively (Pretsch *et al.*, 1988) as expected for an X-linked defect. The animals were crossbred with mice of the C3H strain, homozygous for normal levels of G6PD, to produce female heterozygous C3H × GPDX mice. All animals were maintained in our own Animal Unit and fed normal Rat and Mouse Breeding Diet (Pilsbury's Ltd., Edgbaston, UK) and tap water ad libitum, unless otherwise stated

Correspondence: G.A. Thomas.
Received 19 June 1990; and in revised form 5 October 1990.

*Induction of thyroid lesions*

Fifty-six female mice (18 homozygous and 20 heterozygous for deficient G6PD activity, and 18 normal female C3H mice) were given a single intraperitoneal injection of $^{131}$I (specific activity 6–20 mCi $\mu$g$^{-1}$) in 0.1 ml saline at 3 weeks of age. Half of the animals in each group were given 11 $\mu$Ci, and half 23 $\mu$Ci. One week later goitrogen administration in the drinking water was commenced. Mice are relatively resistant to the goitrogenic effect of aminotriazole; we have found that a combination of 0.2% aminotriazole and 0.5% sodium perchlorate, sweetened with 0.5% sucrose is both palatable and a consistently effective goitrogen.

After 46 weeks, half of the mice receiving each radiation dose were killed by $CO_2$ inhalation. The goitrogen regime was withdrawn from the remaining 28, which were given normal tap water for a further period of 4 weeks until sacrifice.

*Assessment of lesions*

Thyroids were snap frozen in dry ice-cooled isopentane and sectioned in a cryostat. Two serial frozen sections (6 $\mu$m) were cut at 100 $\mu$m intervals through the gland, one stained with haematoxylin and eosin and one with the histochemical technique for G6PD. Briefly, sections are incubated in media containing glucose-6-phosphate and NADP as susbtrates. The reaction product of the enzyme is visualised by reduction of nitroblue tetrazolium (see Thomas *et al.*, 1989). Numerous lesions were found, and only those present in the median section through the gland were assessed for quantitation. None showed the features of nodules; all were classified as either adenoma or carcinoma. Clear evidence of extracapsular vascular invasion was present in all the carcinomas.

### Results

No significant difference with respect to number and type of tumours was observed between the groups of mice given 11 $\mu$Ci and those given 23 $\mu$Ci $^{131}$I. There were no intercurrent deaths in either group of animals.

*Animals maintained on goitrogen prior to sacrifice*

Multiple lesions were observed in all animals. A total of 98 lesions were counted (Table I), the average number of lesions per animal being 3.5 ± 2.1 (range 1–11). Of these, 84 showed the morphological characteristics of adenomas – sharply circumscribed or encapsulated lesions, lacking any evidence of invasion. They were cellular tumours with small follicles or trabeculae showing scanty or absent colloid. The tumour

**Table I** Effect of the withdrawal of TSH stimulation on number and type of thyroid lesion

| | Tumour number | |
|---|---|---|
| *Tumour type* | *Goitrogen group*[a] | *Withdrawal group*[b] |
| Adenoma | 84 | 71 |
| Carcinoma | 14* | 2* |
| Total | 98 | 73 |

[a]28 animals maintained on goitrogen for 46 weeks; [b]28 animals from whom goitrogen was withdrawn for four weeks after 46 weeks of treatment; *$\chi^2$: $P < 0.01$.

**Table II** G6PD phenotype of thyroid lesions induced in C3H x GPDX mice after 46 weeks of goitrogen treatment

| *Tumour phenotype* | *Number of adenomas* | *Number of carcinomas* |
|---|---|---|
| Uniformly positive | 19 | 4 |
| Uniformly negative | 13 | 1 |
| Mixed | 0 | 0 |
| Total | 32 | 5 |

**Figure 1a** One large and two small adenomas (A) in an H and E stained thyroid section from a heterozygous C3H x GPDX mouse maintained on goitrogen until sacrifice. A small amount of non-neoplastic, but radiation damaged thyroid tissue (N) can be seen adjacent to the tumours. The size bar represents 100 μ. **b**, Serial section to Figure 1**a** stained with the histochemical technique for G6PD. Each of the adenomas (A) exhibits a single enzyme phenotype, two positive and one negative, consistent with a monoclonal origin. Both cellular phenotypes can be observed in the background non-neoplastic thyroid (N). The size bar represents 100 μm.

**Figure 2** Thyroid follicular carcinoma with extracapsular vascular invasion from a female mouse maintained on goitrogen until sacrifice. H and E stained section. The size bar represents 50 μm.

cells were large with regular vesicular nuclei (Figure 1a). Fourteen carcinomas were observed, these were morphologically similar to the adenomas, but showed capsular invasion and permeation of extracapsular veins (Figure 2). Non-neoplastic epithelium was present as small hyperplastic follicles with the nuclear pleomorphism typical of radiation damage.

*Animals withdrawn from goitrogen treatment 4 weeks prior to sacrifice*

Multiple lesions were again observed in all animals, the average number of lesions per animal being 2.6 ± 1.8, with a range from 1–7. Of a total of 73 counted, 72 were adenomas and two were carcinomas. The decrease in the number of carcinomas observed was statistically significant ($\chi^2$: $P = <0.01$; see Table I).

The decrease in the number of tumours in animals from which goitrogen had been withdrawn was also accompanied by a change in their morphology. The lesions showed a large follicle pattern with abundant colloid, the follicular cells were flattened with small, dark elongated nuclei and scanty cytoplasm. The regressive changes were present in both adenomas and carcinomas and were also seen in the intravascular tumour present in the carcinomas (Figure 3). Interestingly, the marked nuclear pleomorphism characteristic of radiation damage in the background thyroid was unaltered by goitrogen withdrawal.

*Clonality of induced lesions*

G6PD histochemsitry gave a uniform positive staining in the TSH stimulated gland, but considerable intercellular metabolic variation was observed in the non-TSH stimulated gland of mice homozygous for normal levels of the enzyme. (Thomas *et al.*, 1988). For this reason, it is impractical to determine clonality directly in the non-stimulated gland of heterozygous animals. The clonality of lesions in this study was therefore assessed in female heterozygous mice maintained on goitrogen until death.

All lesions observed in each of ten female mice heterozygous for deficient G6PD activity showed uniform expression of a single enzyme phenotype (Figure 1b). Thirty-seven lesions were present in the median sections, five carcinomas and 32 adenomas. Nineteen (59%) adenomas showed a positive enzyme phenotype and 13 (41%) a negative enzyme phenotype. A previous study has predicted that if thyroid tumours were to originate from more than two cells, a minimum of 25% would express a mixed enzyme phenotype (Thomas *et al.*, 1989). The lack of any lesion with a mixed enzyme phenotype confirms that the tumours in this study are of a single cell origin. Four carcinomas expressed a positive enzyme phenotype and one a negative enzyme phenotype, suggesting that they too were monoclonal. Both enzyme phenotypes were clearly separable in the background non-neoplastic thyroid. All lesions in similarly treated homozygous C3H mice showed the expected positive enzyme phenotype, and all lesions in the homozygous GPDX mice the expected negative enzyme phenotype, showing that there was no significant alteration in enzyme expression associated with neoplasia in this study.

a b

**Figure 3a** Thyroid carcinoma induced by radiation and long-term goitrogen treatment showing regression following 4 weeks without goitrogen treatment prior to sacrifice. The lesion in this H and E stained section is composed of colloid-filled follicles lined by flattened, inactive epithelium, instead of the active follicles seen in the tumours shown in Figures **1a** and 2. The size bar represents 50 µm. **b**, Another field from the same carcinoma as **3a**, showing vascular invasion. The thyroid tissue within the extrathyroid vessel shows similar features of regression to the main tumour, demonstrating that invasion is compatable with TSH responsiveness. H and E stained section. The size bar represents 50 µm.

### Discussion

This study has shown that monoclonal thyroid lesions, both adenomas and carcinomas, induced by a mutagen followed by longterm goitrogen treatment regress when the goitrogen is withdrawn. This regression is characterised by morphological changes and by a reduction in the number of tumours observed – a very marked reduction in the case of the carcinomas. Although we have not investigated the reappearance of the malignant phenotype on reintroduction of a high TSH, this has been shown in transplantation experiments (Matovinovic *et al.*, 1970).

The observation of regression in thyroid lesions has previously been interpreted as suggesting that they were hyperplastic rather than neoplastic (Todd, 1986). Jemec (1977) used the term 'seeding of hyperplastic tissue' to describe pulmonary lesions that accumulated colloid when goitrogen was withdrawn. While our own findings of reversion to normal phenotype agree with the earlier work, the additional evidence we have provided showing that these lesions are monoclonal must lead to a different explanation.

We now recognise that although tumours may initially be monoclonal or oligoclonal they progress by clonal selection (Nowell, 1978). While many different steps may be involved during the progression of different thyroid tumours, a small number of key steps are likely to be common to most spontaneous thyroid carcinomas. The normal follicular cell has been shown to possess a growth limiting mechanism (Wynford-Thomas *et al.*, 1982). The continued growth of tumour cells infers that any such mechanism must have been lost in neoplasia. Development of TSH independent growth is also likely to be essential for spontaneous thyroid carcinogenesis. The third key step is the acquisition of the ability to invade – an essential feature of malignancy.

The development of these successive defects that lead to neoplasia can be considered as a process of natural selection at a cellular level; manipulation of the environment in which the selection takes place will alter the selection process. In experimental thyroid carcinogenesis, the induction of persistently high TSH levels creates an environment in which any cell which suffers a mutation (or epimutation) leading to loss of its growth limiting mechanism will give rise to a clone of cells which continue to grow and therefore continue to be at risk of further mutation. As the normal cells continue to be subjected to a maximum TSH stimulation, a mutation leading to the development of TSH independent growth is unlikely to confer any relative growth advantage or increased risk of further mutation on the progeny of that cell. When invasiveness develops in a cell that has not acquired TSH

independent growth, the resulting carcinomas retain TSH dependency and will regress when the TSH stimulation is withdrawn.

The individual mutations required for the development of a carcinoma follicular key steps are not known. They represent three restriction points in the progression of a normal follicular cell to a malignant follicular cell. The first step in the sequence – the loss of the growth limitation mechanism – may require two mutations as mechanisms of the antioncogene type are generally regarded as recessive. It has however recently been recognised that a single hit may lead to the loss of an antioncogene through the synthesis of an altered gene product that blocks the effect of the normal product (Finlay *et al.*, 1989).

A mechanism which may be relevant involves translocation – already known to be important in other tumours, for example in Burkitt's lymphoma (Dalla-Favera *et al.*, 1982) and chronic myelocytic leukaemia (Heisterkamp *et al.*, 1983). For the mechanism to be effective, a growth control gene must be translocated to the region of an active promoter. In the longterm stimulated follicular cell many genes are active, both genes specifically related to thyroid function, like the thyroglobulin gene, and housekeeping genes – as for example G6PD. Prolonged TSH stimulation therefore increases the chance that a translocation may occur to a promotionally active site, with continued expression of the oncogene concerned – but with little or no expression if the TSH stimulation is withdrawn.

We suggest therefore that prolonged TSH stimulation may obviate the need for the development of TSH independent growth in carcinogenesis, and may also increase the chance of excessive TSH dependent growth through translocation to a TSH inducible promoter region. Whether one or both of these mechanisms occurs, the method chosen in experimental carcinogenesis to give a large number of tumours for study may do this by by-passing one stage of the multistep process normally required to generate the infrequent spontaneous carcinomas of the thyroid seen in euthyroid man. Similar principles may underlie models of experimental carcinogenesis in other tissues, and tumours induced in man by prolonged growth stimulation.

### References

DALLA-FAVERA, R., BREGNI, M., ERIKSON, J., PATTERSON, D., GALLO, R.C. & CROCE, C.M. (1982). Human c-myc onc gene is located on the regioin of chromosome 8 that is translocated in Burkitt lymphoma cells. *PNAS USA*, **79**, 7824.

DONIACH, I. (1974). Effects of radiation on thyroid function and structure. In: *Handbook of Physiology*. Vol III Endocrinology. Greer, M.A. & Solomon, D.H., (eds). pp. 359–375.

FINLAY, C.A., HINDS, P.W. & LEVINE, A.J. (1989). The p53 protooncogene can act as a suppressor of transformation. *Cell*, **57**, 1083.

HEISTERKAMP, N., STEPHENSON, J.R., GROFFEN, J. & 4 others (1983). Localisation of the c-abl oncogene adjacent to a translocation breakpoint in chronic myelocytic leukaemia. *Nature*, **306**, 239.

JEMEC, B. (1977). Studies on the tumorigenic effect of two goitrogens. *Cancer*, **40**, 2188.

MATOVINOVIC, J., NISHIYAMA, R.H. & POISSANT, G. (1970). Transplantable thyroid tumors in the rat: development of normal-appearing thyroid follicles in the differentiated tumors and development of differentiated tumors from iodide-deficient, thyroxine-involuted goiters. *Cancer Res.*, **30**, 504.

MORRIS, H.P. & GREEN, C.D. (1951). The role of thiouracil in the induction, growth and transplantability of mouse thyroid tumors. *Science*, **114**, 44.

NOWELL, P.C. (1978). Tumors as clonal proliferation. *Virchows Arch. B. Cell. Path.*, **29**, 145.

PRETSCH, W., CHARLES, D.J. & MERKLE, S. (1988). X-linked glucose-6-phosphate dehydrogenase deficiency in Mus musculus. *Biochem. Genet.*, **1**, 89.

PURVES, H.D., GRIESBACH, W.E. & KENNEDY, T.H. (1951). Studies in experimental goitre: malignant change in a transplantable rat thyroid tumor. *Br. J. Cancer*, **5**, 301.

THOMAS, G.A., WILLIAMS, D. & WILLIAMS, E.D. (1988). The demonstration of tissue clonality by X-linked enzyme histochemistry. *J. Pathol.*, **155**, 101.

THOMAS, G.A., WILLIAMS, D. & WILLIAMS, E.D. (1989). The clonal origin of thyroid nodules and adenomas. *Am. J. Pathol.*, **134**, 141.

THOMAS, G.A., WILLIAMS, E.D. Evidence for and possible mechanisms of non-genotoxic carcinogenesis in the thyroid. *Mutat. Res.* (in press).

TODD, G.C. (1986). Induction and reversibility of thyroid proliferative changes in rats given an antithyroid compound. *Vet. Pathol.*, **23**, 110.

WOLLMAN, S.H. (1963). Production and properties of transplantable tumours in the thyroid gland of the Fischer rat. *Recent Prog, Horm. Res.*, **19**, 579.

WYNFORD-THOMAS, D., STRINGER, B.M.J. & WILLIAMS, E.D. (1982). Dissociation of growth and function in the rat thyroid during prolonged goitrogen administration. *Acta Endocrinol.*, **101**, 210.